# Does obesity have detrimental effects on IVF treatment outcomes?

**DOI:** 10.1186/s12905-015-0223-0

**Published:** 2015-08-19

**Authors:** Murat Ozekinci, Ali Seven, Safak Olgan, Mehmet Sakinci, Ugur Keskin, Munire Erman Akar, Seyit Temel Ceyhan, Ali Ergun

**Affiliations:** Department of Obstetrics and Gynecology, Akdeniz University Faculty of Medicine, 07059 Antalya, Turkey; Department of Obstetrics and Gynecology, Gulhane Military Academy, 06018 Ankara, Turkey

## Abstract

**Background:**

The aim of this study was to investigate the influence of body mass index (BMI) on the in vitro fertilization (IVF) treatment outcomes in a cohort of women undergoing their first IVF, using an intracytoplasmic sperm injection (ICSI).

**Methods:**

This retrospective cohort study included 298 cycles from women younger than 38 years old undergoing IVF-ICSI at a university infertility clinic. The treatment cycles were divided into three groups according to the BMI of the women involved: normal weight (18.5 ≤ BMI < 25 kg/m^2^, 164 cycles), overweight (25 ≤ BMI < 30 kg/m^2^, 70 cycles), and obese (BMI ≥ 30 kg/m^2^, 64 cycles). The underweight women (BMI < 18.5 kg/m^2^) were not included in the analysis due to small sample size (*n* = 22). The patient characteristics and IVF-ICSI treatment outcomes were compared between the BMI groups.

**Results:**

The total gonadotropin dose (*p* <0.001) and duration of stimulation (*p* = 0.008) were significantly higher in the obese group when compared to the normal BMI group. There were no significant differences across the BMI categories for the other IVF-ICSI cycle outcomes measured, including the number of retrieved oocytes, mature oocytes, embryos suitable for transfer, proportion of oocytes fertilized, and cycle cancellation rates (*p* >0.05 for each). Additionally, clinical pregnancy, spontaneous abortion, and the ongoing pregnancy rates per transfer were found to be comparable between the normal weight, overweight, and obese women (*p* >0.05 for each).

**Conclusion:**

Obese women might require a significantly higher dose of gonadotropins and longer stimulation durations, without greatly affecting the pregnancy outcomes.

## Background

Obesity has become a worldwide epidemic, with approximately 1.6 billion adults being overweight and 400 million being obese [1]. Obesity has several serious consequences on health, including hypertension, diabetes mellitus, chronic heart disease, lipid disorders, uterine cancer, and breast cancer [2, 3]. Moreover, obesity has negative effects on reproductive health. It has been established that obesity is associated with decreased natural fecundity, a decreased ovulation rate, increased time until conception, and increased rates of miscarriage [4, 5]. Additionally, an increased rate of pregnancy complications, including gestational hypertension, preeclampsia, gestational diabetes, postpartum hemorrhage, and fetal macrosomia, are all associated with obesity [6, 7]. Since the incidence of obesity is continually rising, an increasing number of overweight and obese women are seeking fertility treatments through assisted reproduction technology (ART) [8]. Consequently, there is a need to understand the full impact of obesity on in vitro fertilization (IVF) treatments.

There is conflicting evidence with regard to the effects of a raised body mass index (BMI) on the outcome of ART. Although some studies have reported no adverse effects of a raised BMI on IVF outcomes [9, 10], others have linked various negative impacts, including a higher dose of gonadotropin stimulation, longer stimulation duration, lower number of retrieved and mature oocytes, and decreased embryo quality [4, 11]. The aim of this study was to investigate the influence of BMI on the outcomes of ART in a cohort of women (≤38 years of age) undergoing their first IVF-ICSI.

## Methods

Between January of 2008 and October of 2013, a total of 1620 IVF-ICSI cycles were carried out at the infertility clinic of the Gulhane Military Academy. Records of the subjects following their first IVF-ICSI cycles were analyzed, and of these, 320 embryo transfer cycles fulfilled our inclusion criteria: (i) women ≤ 38 years of age, (ii) cycles arising from single embryo transfer (SET) or double embryo transfer (DET), and (iii) where the data on the outcome was available. Cycles arising from more than two embryo transfers (ETs) and cryo-thaw embryos were excluded from the analyses.

This study was approved by the institutional review board of the Gulhane Military Academy (approval number 2013/168.4-2543). Since this was a retrospective cohort study, no written informed consent for participation was obtained from the participants. The patients underwent IVF according to standard stimulation protocols, involving pituitary down-regulation with either a GnRH agonist administered in the mid-luteal phase of the prior cycle (long protocol), or a diluted GnRH agonist given on days 2–4 of the cycle (micro-dose protocol). Alternatively, a short protocol with a GnRH antagonist was started when the leading follicle reached 14 mm. Controlled ovarian stimulation was achieved with hMG and/or recombinant FSH. The response to stimulation was monitored using serum E2 and a transvaginal ultrasound. Human chorionic gonadotropin (hCG) was administered to stimulate the final stages of follicular development when the follicles reached maturity, defined by two leading follicles over 18 mm. A transvaginal follicle aspiration was performed 36 h after hCG administration, and the embryo transfer took place 48 h later. Micronized vaginal progesterone, at daily dose of 600 mg, was used for 2 weeks for luteal support.

The embryos were transferred to the uterus either 3 days (cleavage stage) or 5 days (blastocyst stage) after the oocyte retrieval, and the embryo quality was assessed on the day of transfer. The cleavage stage embryos were scored based on cell number and the degree of fragmentation, according to the grading system of Hardarson (2001) [12]. The embryos at an appropriate developmental stage, with <20 % fragments and a mild degree of uneven-sized blastomeres (grade I, grade IIA, and grade IIB), constituted the day-3 embryos that were suitable for transfer. In the case of extended culture, all of the blastocyst stage embryos were evaluated using the grading system of Gardner and Schoolcraft (1994). The blastocysts were graded according to the degree of expansion, and the quality of the inner cell mass and trophoectoderm [13]. The grade III–VI embryos, according to blastocyst expansion and hatching status, with an inner cell mass and trophoectoderm grades of A or B constituted the day-5 embryos suitable for transfer. The total number of embryos suitable for ET (either at the cleavage or blastocyst stage) was calculated for each patient.

At day 12 of the ET, each patient had her *β*-hCG levels assessed. Pregnancy was defined by serially increasing serum *β*-hCG titres to at least 25 IU/l, within 12 days after the cleavage stage ET. All of the patients underwent transvaginal ultrasounds at 5 to 6 weeks of gestation, or when the *β-*hCG exceeded 2,000 IU/L, in order to determine the location and number of the pregnancies. Biochemical pregnancy was defined as a transient pregnancy that spontaneously resolved before the ultrasonographic confirmation. Clinical pregnancy was documented by ultrasonographic evidence of the fetal cardiac activity at 6–7 weeks of gestation. Spontaneous abortion was defined as the loss of a clinical pregnancy. Additionally, ongoing pregnancy was defined as progression beyond 12 weeks of gestation.

The patients’ age, infertility duration-etiology, antral follicle count, baseline FSH, LH, and estradiol levels (cycle day 3) were reviewed. The IVF-ICSI cycle characteristics, including the total gonadotropin dose used, duration of stimulation, number of retrieved oocytes, metaphase 2 (MII) oocytes, proportion of oocytes fertilized, and embryo quality were noted. The women were assessed for polycystic ovary syndrome (PCOS) as defined by the 2003 Rotterdam European Society for Human Reproduction and Embryology/American Society for Reproductive Medicine sponsored PCOS consensus workshop group [14]. The BMI was calculated by using the formula weight/height^2^ from the medical records. According to the World Health Organization (WHO) guidelines, the women were grouped into three categories, namely normal: 18.5–24.9 kg/m^2^; overweight: 25–29.9 kg/m^2^; obese: ≥30 kg/m^2^. Because the number of women in underweight group (BMI <18.5 kg/m^2^) was low (*n* = 22), these patients were excluded from the analysis.

The data were analyzed using the Microsoft Statistical Package for the Social Sciences (SPSS) for Windows, version 22.0. The Kruskal-Wallis test was conducted to explore the differences among the BMI categories, while the Mann-Whitney *U* test was conducted to analyze the continuous and discrete ordinal variables, and the nominal data was analyzed using the *χ*^2^ test. All of the Mann-Whitney U and *χ*^2^ tests were two tailed, and Bonferroni’s correction was used to adjust for multiple comparisons, unless otherwise stated. The potential correlation between the BMI, total gonadotropin dose, stimulation duration, and IVF-ICSI treatment outcomes was evaluated using the Spearman Rank Order Correlation. Finally, multiple regression analyses were used to assess the variables for the total gonadotropin dose and stimulation duration. Preliminary analyses were conducted to ensure no violation of the assumptions of normality, linearity, multicollinearity, and homoscedasticity. A *P* value of <0.05 was considered to be statistically significant.

## Results

In total, 298 consecutive cycles (among 298 women) with SET or DET were subjected to analysis. The mean age at treatment was 30.4 years old (range 21–38), and the clinical characteristics of the patients according to the BMI categories are shown in Table 1. There were no significant differences in the age and smoking status of the women among the BMI categories. The duration of infertility in years was significantly longer in the obese women, when compared to the normal patients (*p* <0.001). However, the duration of infertility was found to be comparable between the overweight and normal/obese patients. The main cause of infertility was unexplained in 71 patients (23.8 %), with the male factor in 67 (22.5 %), anovulation in 31 (10.4 %), tubal factor in 63 (21.1 %), poor ovarian reserve in 12 (4.0 %), endometriosis in 12 (4.0 %), and combined causes in 42 (14.1 %) patients. The distributions of the etiologies were found to be similar between the BMI categories. With regard to the clinical characteristics, there were no significant differences in the total number of antral follicles, polycystic ovaries upon ultrasound, PCOS, or the baseline FSH, LH, and estradiol levels between the groups.

**Table 1:** Characteristics of the patients according to BMI

	BMI			
	18.5–24.9	25.0–29.9	≥30.0	*P*
	(*n* = 164)	(*n* = 70)	(*n* = 64)	
BMI (kg/m^2^)	21.9 ± 1.7^a^	27.1 ± 1.4^b^	33.1 ± 2.2^c^	<0.001
Women’s age (yr)	30.1 ± 3.9	30.8 ± 3.8	30.8 ± 4.4	0.313
Years of infertility	4.1 ± 3.1^a^	4.7 ± 2.8^a,b^	5.7 ± 2.9^b^	0.002
Ever pregnant prior to inclusion	15 (9)	7 (10)	4 (8)	0.443
Smoking	44 (23.7)	18 (25.7)	13 (20.3)	0.106
Baseline FSH (pg/mL)	6.3 ± 1.7	6.2 ± 1.8	6.2 ± 1.6	0.550
Baseline LH (pg/mL)	6.1 ± 2.8	5.9 ± 1.8	4.8 ± 2.7	0.262
Baseline estradiol (pg/mL)	52.8 ± 26.6	50.7 ± 22.2	58.6 ± 27.1	0.193
Antral follicle count	10.7 ± 6.0	10.9 ± 6.6	12.4 ± 6.9	0.206
Polycystic ovaries on ultrasound	22 (13.4)	11 (15.7)	13 (20.3)	0.874
Polycystic ovary syndrome^*^	35 (26.9)	16 (29.1)	16 (31.4)	0.087
Unexplained infertility	41 (25.0)	16 (22.9)	14 (21.9)	0.657
Poor ovarian reserve	6 (3.7)	3 (4.3 %)	3 (4.7)	0.974
Male factor	38 (23.2)	16 (22.9)	13 (20.3)	0.858
Tubal Factor	34 (20.7)	16 (22.9)	13 (20.3)	0.692
Endometriosis	5 (3.0)	3 (4.3)	4 (6.3)	0.322
Anovulation	15 (9.1)	8 (11.4)	8 (12.5)	0.128
Combined infertility	25 (15.2)	8 (11.4)	9 (14.1)	0.332

Data are presented as mean ± standard deviation or n (%). *BMI* body mass index, *ns* non significant (*p* <0.05)

Values across a row with different superscripts (^a-c^) indicate significant difference between pregnancy outcome categories (*p* <0.017), and values across an individual row with matching superscripts (^a-c^)indicate no significant difference between pregnancy outcome categories. ^*^Excluding 62 cases (34 in normal-weight, 15 in overweight, and 13 in obese group) in which the complete data could not be obtained from the records available

**Fig. 1:**
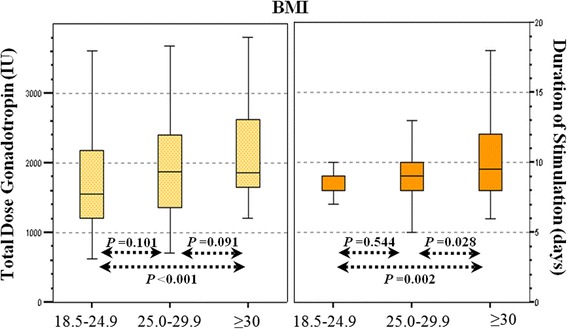
Box-plots indicating total gonadotropin dose (IU) and duration of stimulation (days) among BMI categories. The medians and 25th-75th percentiles are shown. Mann-Whitney *U* test was used for the analysis

Regarding the IVF-ICSI treatment outcomes, the total gonadotropin dose (*p* <0.001) and duration of stimulation (*p* = 0.008) were found to be significantly different across the BMI categories. Subsequently, the total gonadotropin dose (*p* <0.001) and duration of stimulation (*p* = 0.002) were significantly different between the normal and obese patients (Table 2, Fig. 1). However, the gonadotropin dose and stimulation duration were found to be comparable in the overweight and normal, and overweight and obese patients. Additionally, there were no significant differences between the BMI categories in the number of retrieved oocytes, number of MII oocytes, proportion of oocytes fertilized, number of embryos suitable for transfer, and number of canceled cycles. The number of transferred embryos was found to be similar between the BMI categories (SET was performed in 81.8 %, 79.4 %, and 78.0 % of the patients in the normal, overweight, and obese patients, respectively). The percentage of blastocyst transfers was found to be comparable between the groups (embryo culture was extended in 34.6 %, 39.1 %, and 33.9 % of the patients in the normal, overweight, and obese patients, respectively). Finally, the pregnancy outcomes, including the implantation rate, biochemical, ectopic, clinical, and ongoing pregnancy, and spontaneous abortion (per transfer) were found to be similar between the BMI categories.

**Table 2:** Characteristics of IVF-ICSI treatment according to body mass index (BMI)

		BMI			
		18.5–24.9	25.0–29.9	≥30.0	*P*
		(*n* = 164)	(*n* = 70)	(*n* = 64)	
Gonadotropin dose,					
Total for cycle (IU)		1859 ± 1065^a^	2015 ± 968^a,b^	2455 ± 1343^b^	<0.001
Per retrieved oocyte (IU)		256 ± 429	249 ± 336	249 ± 192	0.112
Per M2 oocyte (IU)		338 ± 557^a^	313 ± 358^a^	436 ± 434^b^	0.017
Per embryo suitable for transfer (IU)		785 ± 1039	661 ± 562	806 ± 749	0.087
Duration of stimulation (days)		8.7 ± 1.8^a^	8.9 ± 1.9^a,b^	9.8 ± 2.4^b^	0.008
Ovarian stimulation protocol					
Long protocol		99 (60.4)	39 (55.7)	36 (56.2)	0.334
Short (antagonist) protocol		55 (33.5)	26 (37.2)	24 (37.5)	0.256
Micro dose protocol		10 (6.1)	5 (7.1)	4 (6.3)	0.134
No. of retrieved oocytes		12.3 ± 6.2	12.8 ± 6.1	13.2 ± 7.3	0.821
No. of MII oocytes		9.5 ± 5.3	9.2 ± 5.4	9.0 ± 6.9	0.329
Proportion of oocytes fertilized (%)		75.6 ± 20.1	79.1 ± 20.3	75.4 ± 22.1	0.215
No. of embryos suitable for transfer		4.2 ± 3.0	4.5 ± 3.3	3.6 ± 2.9	0.160
Extended culture		55 (34.6)	27 (39.1)	20 (33.9)	0.591
Canceled cycles		5 (3.0)	2 (2.9)	5 (7.8)	0.119
No. of transferred embryos	SET	130 (81.8)	54 (79.4)	46 (78.0)	0.416
	DET	29 (18.2)	14 (20.6)	13 (22.0)	
Pregnancy outcome^*^					
Implantation		62 (39.0)	26 (38.2)	21 (35.6)	0.755
Biochemical pregnancy		6 (3.8)	4 (5.8)	1 (1.7)	0.483
Ectopic pregnancy		2 (1.3)	0 (0.0)	1 (1.7)	0.595
Clinical pregnancy		52 (32.7)	22 (32.4)	18 (30.5)	0.953
Spontaneous abortion		6 (3.8)	3 (4.3)	3 (5.1)	0.909
Ongoing pregnancy		46 (28.9)	19 (27.9)	15 (25.4)	0.874

Data are presented as mean ± standard deviation or n (%). *BMI* body mass index, *MII* metaphase 2, *SET* single embryo transfer, *DET* double embryo transfer, *ns* non significant (*p* <0.05)

Values across a row with different superscripts (^a-b^)indicate significant difference between pregnancy outcome categories (*p* <0.017), and values across an individual row with matching superscripts (^a-b^)indicate no significant difference between pregnancy outcome categories. ^*^Data for pregnancy outcomes are expressed per embryo transfer

The relationship between the BMI and IVF-ICSI treatment outcomes was investigated using the Spearman rank order correlation coefficient. There were positive weak correlations between the BMI and the total gonadotropin dose (*r* = 0.27, *p* <0.001), and the duration of stimulation (*r* = 0.20, *p* = 0.001). Additionally, there were no statistically significant correlations between the BMI and the number of retrieved oocytes, number of MII oocytes, proportion of oocytes fertilized, number of embryos suitable for transfer, spontaneous abortion, and the ongoing pregnancy rates (*p* <0.05).

The multiple regression analysis was used to assess the ability of the BMI to predict the total gonadotropin dose and duration of stimulation (Table 3). The age, duration of infertility, and presence of PCOS were added into the same model, since they are potentially confounding factors. The prediction model was statistically significant (*p* <0.001), and accounted for approximately 10 % of the variance in the total gonadotropin dose (adjusted R2 = 0.10). In the final model, the age (*p* = 0.001) and BMI (*p* = 0.043) were found to be statistically significant for predicting the total gonadotropin dose. The corresponding adjusted R2 value for the prediction model of the stimulation duration was 0.07 (*p* <0.001). In this model, the BMI (*p* = 0.007) and PCOS (*p* = 0.008) were found to be statistically significant.

**Table 3:** Multiple regression analysis of variables for total gonadotropin dose and duration of stimulation

Variable	Multiple regression analysis			
	Gonadotropin dose, total for cycle (IU)^a^		Duration of stimulation (days)^b^	
	*β* value	*P* value	*β* value	*P* value
Age (years)	0.316	0.001	0.073	0.259
Years of infertility	−0.025	0.707	0.044	0.498
BMI (kg/m^2^)	0.136	0.043	0.179	0.007
PCOS	0.029	0.663	0.178	0.008

*BMI* body mass index, *PCOS* polycystic ovary syndrome

^a^Adjusted R^2^ = 0.10

^b^Adjusted R^2^ = 0.07

## Discussion

Higher gonadotropin consumption and longer stimulation durations were observed in the obese females, when compared with the normal weight women. However, we did not find any significant differences in terms of embryological parameters and cycle cancelation rates across the BMI categories. Similarly, clinical pregnancy, spontaneous abortion and ongoing pregnancy rates were found to be comparable between the groups.

The duration of infertility was significantly higher in the obese women, when compared to the normal weight women, although the mean age of the women between the BMI categories was similar. Since it was the first fresh cycle for each woman, this might indicate a possible delay in the infertility diagnosis or the lack of a woman’s desire to seek treatment. We believe that multiple etiologies, including the concomitant health consequences or psychological factors that are attributable to obesity, might play a role in this issue.

In the current study, trends of higher gonadotropin consumption and longer stimulation durations were noted in the obese females, when compared with the normal weight women. Similar to our results, a higher dose of gonadotropin stimulation and a longer stimulation duration were reported in the women with BMIs = 25–29.9 kg/m^2^, when compared with the women with normal BMIs [15, 16]. Additionally, it was also shown that a higher dose of gonadotropin stimulation and longer stimulation duration were required in women with BMIs >30 kg/m^2^, when compared with women having normal BMIs [15, 16]. Nevertheless, Farhi et al. reported no statistically significant differences in the dose of gonadotropin stimulation in women with BMIs ≥25 kg/m^2^, compared with women with BMIs <25 kg/m^2^ [17]. Therefore, an increase in the gonadotropin dose and stimulation duration might be more prominent in obese than overweight patients; however, the majority of studies in this context do not discriminate isolated obesity from the patients with PCOS. Since PCOS is frequently associated with an increased BMI, it might be essential to evaluate those patients independently, in order to delineate the impact of isolated obesity on IVF-ICSI outcomes. In order to address this question, a multiple regression analysis was performed, including both the BMI and PCOS. Similar to our results, Loh et al. (2002) reported a significant increase in the gonadotropin dose and duration of stimulation, with BMIs >30 kg/m^2^ in non-PCOS patients [18].

With regard to the IVF-ICSI treatment outcomes, we did not find any significant differences in the number of retrieved oocytes, number of MII oocytes, proportion of oocytes fertilized, embryo grades, and cycle cancelation across the BMI categories. Currently, the literature lacks consensus on the effects of obesity on IVF-ICSI treatment outcomes. Although some studies have revealed a lower number of oocytes retrieved in overweight and obese women [10, 15], other studies were not able to show such a difference in the number of oocytes retrieved among the different BMI groups [19–21]. Another concern is whether obesity affects oocyte maturity or not. Dokras et al. have shown that the number of mature oocytes was significantly reduced in morbidly obese women [20]; whereas, Metwally et al. have shown that obesity did not have any significant effects on the oocyte quality [22]. Studies have also assessed the impact of the BMI on the fertilization rate. Matalliotakis et al. recently found a significantly reduced fertilization rate in women with BMIs of at least 24 kg/m^2^, when compared with those with BMIs of less than 24 kg/m^2^ (58.9 vs. 51.7 %) [10]. However, most of the other studies did not report a significant difference in the fertilization rates between the obese or overweight women and the normal women [15, 20, 21]. Likewise, some studies have reported decreased embryo quality in obese and overweight women [23], but the majority of studies, again, did not show any significant effects with regard to this issue [24, 25]. Consequently, there is largely conflicting evidence regarding the effects of a raised BMI on the IVF-ICSI outcomes.

Rittenberg et al. recently conducted a meta-analysis in order to explore the effects of the BMI on IVF treatment outcomes [8]. They found significant reductions in the clinical pregnancies and an increase in the miscarriage rates in the women with BMIs of 25–29.9 kg/m^2^ and ≥30 kg/m^2^, when compared to the women with normal BMIs. Although we observed a decreasing trend in the clinical and ongoing pregnancy rates in the normal through obese women, the differences did not reach statistical significance in our study. Similarly, the spontaneous abortion rates were found to be comparable between the groups.

We analyzed only the first fresh ART cycle of every patient who met the inclusion criteria, to minimize the confusing effects caused by the history of repeatedly failed or cryo-preserved cycles. Additionally, in women older than 38 years of age, which may specifically affect the duration of ovarian stimulation, gonadotropin dose and cancellation, and the implantation rates, were excluded from the analyses. Despite these measures, our study has several limitations, mainly due to its retrospective nature (e.g., missing data), as well as its relatively small sample size and difficulty in controlling the prior dose, type, and overall amount of gonadotropin administered. The gonadotropin starting dose is usually chosen according to the woman’s age, BMI, and markers of ovarian reserve, including the antral follicle count. However, we were unable to measure the anti-mullerian hormone (AMH) levels at our institution, which might complicate the evaluation of the association between the total gonadotropin dose and the BMI. In addition, the evaluation of the obesity in a cohort of patients, excluding the poor responders, would be still appropriate. Furthermore, prospective investigations with a larger sample size of obese patients (considering abdominal obesity, adipokines and uterine environment) and more BMI cut-off points might be necessary to intrinsically explore the effects of obesity on women’s reproduction potential with regard to ART.

## Conclusion

Our findings indicate that obese women might require significantly higher doses of gonadotropins, and longer stimulation durations, without greatly affecting the pregnancy outcomes.
